# Wnt Signaling in the Development of Bone Metastasis

**DOI:** 10.3390/cells11233934

**Published:** 2022-12-05

**Authors:** Noa Ben-Ghedalia-Peled, Razi Vago

**Affiliations:** Avram and Stella Goldstein-Goren Department of Biotechnology Engineering, Ben-Gurion University of the Negev, P.O. Box 653, Beer Sheva 8410501, Israel

**Keywords:** Wnt signaling, β-catenin, cancer, bone metastasis, osteolysis, osteogenesis

## Abstract

Wnt signaling occurs through evolutionarily conserved pathways that affect cellular proliferation and fate decisions during development and tissue maintenance. Alterations in these highly regulated pathways, however, play pivotal roles in various malignancies, promoting cancer initiation, growth and metastasis and the development of drug resistance. The ability of cancer cells to metastasize is the primary cause of cancer mortality. Bone is one of the most frequent sites of metastases that generally arise from breast, prostate, lung, melanoma or kidney cancer. Upon their arrival to the bone, cancer cells can enter a long-term dormancy period, from which they can be reactivated, but can rarely be cured. The activation of Wnt signaling during the bone metastasis process was found to enhance proliferation, induce the epithelial-to-mesenchymal transition, promote the modulation of the extracellular matrix, enhance angiogenesis and immune tolerance and metastasize and thrive in the bone. Due to the complexity of Wnt pathways and of the landscape of this mineralized tissue, Wnt function during metastatic progression within bone is not yet fully understood. Therefore, we believe that a better understanding of these pathways and their roles in the development of bone metastasis could improve our understanding of the disease and may constitute fertile ground for potential therapeutics.

## 1. Wnt Signaling

Wnt signaling occurs through evolutionarily conserved pathways that regulate pivotal processes during embryogenesis and postnatal development [1]. These pathways are activated by the binding of Wnt ligands in a paracrine or autocrine manner to the Frizzled (Fz) receptor and other co-receptors, thereby stimulating different intracellular signaling cascades [2,3]. The two main groups of Wnt signaling pathways comprise the canonical pathway, also termed the β-catenin-dependent pathway, and the non-canonical or β-catenin-independent pathways, which include the planar cell polarity pathway (PCP), the Wnt/calcium pathway and other non-canonical Wnt pathways [4,5]. Nineteen Wnt ligands have been identified in vertebrates, including the typical canonical Wnt ligands Wnt3, Wnt3a, Wnt7a, Wnt8 and Wnt10 and non-canonical ligands such as Wnt4, Wnt5a, Wnt5b and Wnt11, with additional ones that are inconclusive [6]. The presence and expression of different Wnt ligands, Fz receptors, co-activators and domains are dependent on the cell type, environment and receptor milieu [2,7]. The activation of Wnt results in the proliferation, differentiation, motility, polarity, apoptosis, survival, adhesion and fate decisions of stem cells [1]. The correct functioning of these processes depends on tight regulation that is provided by a variety of extracellular and intracellular antagonists and inhibitors, different enhancers, and crosstalk with other signaling pathways [3,8,9,10,11]. Dysfunction of the Wnt pathway, which typically results in birth defect disorders, also occurs during cancer development and progression [12,13,14,15]. Some studies demonstrated the complementary roles of canonical and non-canonical Wnt signaling pathways [4,7,16,17]. The canonical pathway usually takes place in a self-renewing and undifferentiated state, which is stimulated by the exogenous application of Wnt3a to cell cultures and by LRP5 co-receptor overexpression [10,18,19]. The non-canonical Wnt5 ligands enhance differentiation and migration and curtail proliferation [20,21].

### 1.1. Canonical Wnt Signaling

**Figure 1:**
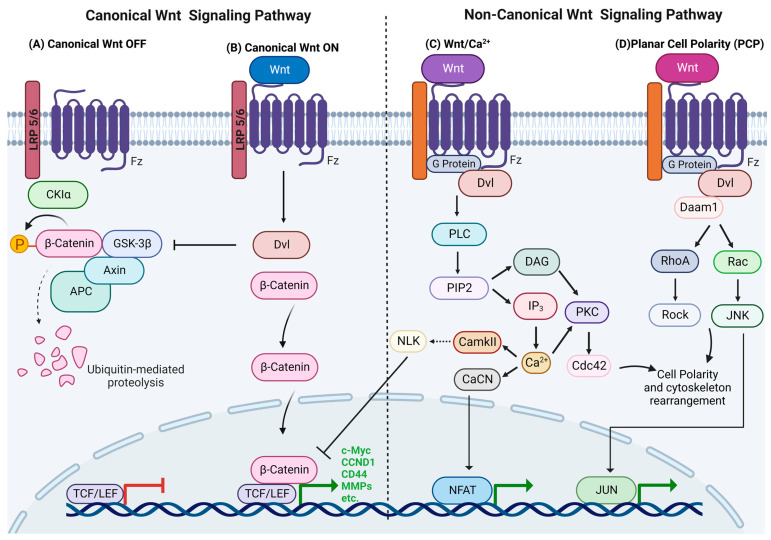
Overview of Wnt signaling pathways. (**A**) Canonical Wnt pathway off. (**B**) Canonical Wnt pathway on. (**C**) Non-canonical Wnt/Ca^2+^ pathway and (**D**) non-canonical Wnt/PCP pathway.

The canonical Wnt pathway is also termed β-catenin-dependent Wnt due to its role in the stabilization of β-catenin. In the absence of Wnt ligands, intracellular levels of β-catenin remain low due to its degradation by the β-catenin destruction complex, which consists of Axin, CK1α, APC and GSK3β. The destruction complex facilitates β-catenin phosphorylation by CKIα and GSK3β, targeting it for ubiquitination and subsequent proteolytic destruction by the proteasomal machinery by using β-TrCP [22] (Figure 1A). The canonical Wnt pathway is activated by Wnt ligands binding to the Fz and LRP5/6 co-receptors, and it triggers a series of events that disrupt the APC/Axin/GSK3β complex. LRP5/6 phosphorylation by CKIγ or GSK3 induces the membrane translocation and binding of Axin and Dishevelled (Dvl) to LRP5/6. Fz undergoes phosphorylation and becomes activated, while Axin is dephosphorylated and degraded. Activated Dvl inhibits GSK3′s activity and activates a series of events that prevent β-catenin degradation, which causes its stabilization and accumulation in the cytoplasm, after which it translocates into the nucleus and participates in genetic transcription along with other transcription factors, such as the TCF/LEF DNA-binding transcription factors, and their target genes are c-Myc, cyclin D1, CD44, metalloproteinases, etc. [1,2,11] (Figure 1B). The c-Myc oncogene is a transcription factor that drives cell cycle progression and proliferation by upregulating cyclins, such as cyclin D1, and that reduces apoptosis [23].

### 1.2. Non-Canonical Wnt Signaling

Non-canonical Wnt signaling includes all of the pathways that are activated by Wnt ligands that are β-catenin independent. In contrast to canonical Wnt signaling, these pathways are not well understood [24]. The main roles of the two principal non-canonical Wnt pathways, the calcium dependent Wnt and PCP pathways, comprise cytoskeletal remodeling, transcriptional regulation and migration [1,3].

#### 1.2.1. Wnt/Calcium Pathway

The main impact of this pathway is increased cell cytoplasmic levels of Ca^2+^, which can act as a second mediator in signal transduction via its activation of several Ca^2+^-sensitive proteins [25]. Wnt ligands bind to the receptor complex, including the co-receptor, receptor tyrosine kinase-like orphan receptor 2 (ROR2), and it stimulates heterotrimeric G-proteins and Dvl, which activate phospholipase C (PLC) or cGMP-specific PDE. Activated PLC cleaves the plasma membrane component PIP2 into DAG and IP3, and the subsequent binding of IP3 to receptors on the membrane of the endoplasmic reticulum (ER) opens Ca^2+^ channels to enable calcium release. DAG and the increased Ca^2+^ concentration can activate PKC, which activates a small GTPase, Cdc42, that regulates actin cytoskeleton remodeling and cell migration. The available Ca^2+^ combines with calmodulin to activate phosphatase calcineurin (CaCN) and Ca^2+^/calmodulin-dependent protein kinase II (CaMKII). CaCN activates the transcription factor nuclear factor of activated T cells (NFAT) by dephosphorylation, and NFAT then migrates to the nucleus, where it alters gene expression that regulates cell adhesion and migration and tissue separation. CaMKII, in turn, activates TAK1, and the latter then activates nemo-like kinase (NLK), which phosphorylates TCF and inhibits TCF/ß-catenin-dependent transcription [26,27,28] (Figure 1C). Ling et al. (2009) showed that the non-canonical Wnt5a ligand can inhibit the transcriptional activity of the canonical Wnt pathway, thereby reducing the MSC proliferation rate [22].

#### 1.2.2. Planar Cell Polarity (PCP)

The PCP pathway regulates actin cytoskeleton rearrangement and directs cellular polarity and morphology, thus contributing to cell and tissue organization [29,30]. Wnt interaction leads to signal transduction to Dvl, and its PDZ and DEP domains activate two parallel pathways that, in turn, activate the small GTPases Rho and Rac. The Rho pathway is initiated when Wnt induces the formation of the Dvl-Daam1 complex, which activates the Dvl-associated activator of morphogenesis 1 (Daam1), and Daam1 activates Rho GTPase by the Rho guanine exchange factor WGEF. Rho GTPase activation leads to the activation of Rho-associated kinase (ROCK) and myosin, which leads to the modification of the actin cytoskeleton and cytoskeletal rearrangement [29,30,31,32]. Daam1 also mediates actin polymerization via the binding of Profilin to actin. The Rac pathway starts with the activation of Dvl, whose DEP domain activates Rac GTPase, which stimulates c-Jun N-terminal kinase (JNK) activity (Figure 1D). The Rho and Rac pathways play roles in cytoskeletal modulation, cellular polarity and transcriptional regulation, as well as cell morphology and migration. PKA is a negative regulator of non-canonical signaling that interacts with RhoA and inhibits Rho activity [3,33].

## 2. Wnt in Bone Development and Maintenance

As mentioned above, Wnt signaling is a key regulator of developmental and postnatal processes [1,2]. One such process is postnatal bone homeostasis regulation, which is maintained by the delicate balance between bone formation and resorption, which are controlled by the dynamics between bone cells. The three main cellular players involved in bone homeostasis comprise one of hematopoietic origin, the osteoclasts, responsible for bone resorption, while the others are of mesenchymal origin. These comprise osteoblasts, which are responsible for bone mineralization and formation, and osteocytes, which are derived from osteoblasts and responsible for bone maintenance by regulating the activities of osteoclasts and osteoblasts. Osteocytes are mechanosensory cells that can produce different molecules and transmit signals in response to physical forces [34,35]. In recent years, the role of Wnt signaling in bone biology has become evident, and there is an increased understanding that aberrant Wnt signaling activity/regulation may cause different bone pathologies [34,36]. Canonical Wnt ligands have been found to promote the differentiation of mesenchymal stem cells (MSCs) into the osteoblast lineage and to inhibit chondrogenic and adipogenic cell fates [35,37]. The activity of this pathway in mature osteoblasts leads to their terminal differentiation into osteocytes and regulates the apoptosis of osteoblastic cells [38]. Moreover, Wnt-β catenin pathway activity in osteoblasts and osteocytes upregulates the expression of the osteoclastogenesis inhibitory factor osteoprotegerin (OPG), which binds to RANKL and inhibits the RANK-RANKL interaction, eventually reducing osteoclastic activity and hampering bone resorption [34,36,39]. Canonical Wnt pathway activity in bone thus leads to increased bone mass, and Wnt inhibition may cause a decrease in bone mass. For example, Bennett et al. showed that Wnt10b facilitates osteogenesis via the expression of RUNX2 and Dlx5, osteoblastogenic transcription factors, and that it increases bone mass in transgenic mice [40]. In addition, in vitro studies showed that different Wnt ligands, such as Wnt 1, 2, 3, 6, 7 and 10, promote osteogenesis and inhibit chondrogenesis and adipogenesis via the canonical Wnt pathway [36,41]. On the other hand, loss-of-function mutations in the canonical Wnt co-receptors LRP5/6 have been identified as a cause of osteoporosis pseudoglioma syndrome [39]. In addition, the aberrant regulation of Wnt inhibitors such as Sclerostin (SOST) and Dickkopf (DKK), which bind to LRP5/6, was found to cause different bone diseases, such as sclerosteosis and osteoporosis [42]. Though SOST dysregulation was identified as the gene responsible for sclerosteosis, its upregulation suppresses bone formation via canonical Wnt inhibition [34,39]. Recently, a SOST inhibition agent was introduced as a new osteoporosis therapy [43,44]. In other research, mice in which the Wnt ligand secretory system was inhibited were shown to have increased bone resorption and diminished bone formation that led to severe osteoporosis [45].

As described above, the role of canonical Wnt signaling in bone formation and resorption has been extensively investigated. Less well understood, however, are the roles that the non-canonical Wnt pathways fulfill in bone homeostasis and the crosstalk between the pathways, but work in recent years has made some progress. Liu et al. found that the non-canonical Wnt receptor ROR2 promotes osteoblast differentiation via the expression of osterix, an osteogenic transcription factor, resulting in bone formation [46]. In addition, the binding of Wnt5a, a non-canonical Wnt ligand, to ROR2 activates JNK, regulates the expression of RUNX2 and inhibits adipogenesis through the repression of the transcription factor PPAR-γ [34,47]. Yu et al. showed that the expression of a non-canonical Wnt ligand, Wnt4, in osteoblasts correlates with a high-bone-mass phenotype [48]. Non-canonical Wnt was found to have a role in osteoclast differentiation and function. Maeda et al. demonstrated that non-canonical Wnt promotes the expression of RANK, resulting in osteoclastogenesis and bone resorption [49]. Wnt16, however, was shown to activate the non-canonical pathway and to inhibit osteoclast differentiation through the suppression of RANKL [50]. Nevertheless, all branches of Wnt signaling, the crosstalk and the delicate balance between all of the Wnt components, are essential for the regulation of bone homeostasis and proper bone remodeling, However, a better understanding of the crosstalk between the pathways is needed.

## 3. Bone Metastasis

Metastasis is the primary cause of mortality in patients with cancer. During cancer development, cancer cells detach from the primary tumor and penetrate the nearby blood and lymphatic vasculature, a process that is called intravasation. From there, they circulate in the blood or lymphatic system while evading the immune system, eventually extravasating the circulation to enter other tissues or organs, where they create a secondary tumor [51]. The ability of cancer cells to metastasize involves changes in adhesion molecules, such as cadherins and integrins that change cell–matrix interactions and the upregulation and activation of extracellular proteases that degrade the extracellular matrix (ECM) and thus facilitate invasion. Termed the “epithelial-mesenchymal transition” (EMT), cancer cells exploit this process to acquire the mesenchymal phenotype that enables them to migrate and invade distant tissues [52,53]. 

Bone constitutes one of the most frequent sites of metastases arising from breast, prostate, lung, thyroid, bladder, melanoma and kidney cancer [54]. This usually occurs in the metaphysis and diaphysis of long bones [55,56,57]. Once cancer cells reach the bone, it can rarely be cured, and therefore, it is a major cause of morbidity associated with severe pain, which can lead to skeletal-related events (SREs), such as impaired mobility, pathologic fractures, hypercalcemia, spinal cord compression and bone marrow aplasia [54]. The common treatment for bone metastases is mainly palliative, including pain control, the prevention and treatment of fractures, and the maintenance of patient function. The efficacy of anticancer drugs is associated with dose-limiting side effects in healthy tissues, and therefore, they do not always achieve therapeutic concentrations. In particular, these limiting factors take place within the complex structure of bone containing dense minerals, ECM and delicate niches that safeguard and control the development of crucial stem cells [58]. The only systemic chemotherapy options for prostate cancer patients are mitotic inhibitors known as taxanes. For patients with breast cancer bone metastasis, treatment relies on chemotherapeutic compounds in the armamentarium, which are inhibitors of DNA synthesis and RNA synthesis, the microtubule disruptor vinorelbine and the thymidylate synthase inhibitor capecitabine [59].

### 3.1. Mechanism of Bone Metastasis

#### 3.1.1. Bone Tropism

Organotropism is a well-known phenomenon in which different tumors preferentially colonize certain organs in a non-random process that cannot be simply explained by blood circulation patterns [60]. To explain this phenomenon, over a century ago, Stephen Paget proposed the “seed and soil” hypothesis, wherein he suggested that the successful colonization of a distant organ is highly dependent on the interplay between the features of the tumor cells, or the “seed”, and the properties of the foreign microenvironment, which represents the “soil” [61]. In skeletal metastases, the bone microenvironment indeed provides fertile ground for metastatic cancer cell growth and survival, as it is characterized by high calcium concentrations, low oxygen levels, high vascularization, and an acidosis environment [62,63,64]. The cells in the bone marrow and the processes of bone formation and resorption release and activate different chemoattractants, i.e., survival and growth factors. These molecules play an important role in the selective homing and retention of disseminated tumor cells (DTCs) in the bone marrow vasculature, and they may contribute to bone metastasis development [54,65]. This unique microenvironment, however, can be a challenge to DTCs, which must adapt to its unfamiliar conditions to survive and proliferate in the bone [63]. As such, to identify effective therapeutics for bone metastases, a better mechanistic understanding of metastatic organotropism is needed. 

#### 3.1.2. Process of Bone Metastasis Development

The multi-step process of bone metastasis development is initiated by the establishment of a pre-metastatic niche at distant organs, DTC penetration of the circulation and evasion of the immune system, followed by their homing and metastatic colonization in the bone. DTCs that survive may grow immediately or enter a dormant state that may last for years, and they can be reactivated at any point in time to create an overt metastasis [66]. The primary tumor secretes different factors—such as CCL2, IL6, lysyl oxidase (LOX) and DKK1—into the circulation to create a pre-metastatic niche in the bone by altering the bone microenvironment [67]. Chemoattractive and adhesion molecules play important roles in the selective homing and retention of cancer cells in the bone marrow vasculature, such as stromal cell-derived factor-1 (SDF-1), a chemokine ligand that binds to CXC chemokine receptors 4 and 7 (CXCR4 and CXCR7). Osteoblasts and other bone marrow stromal cells express this chemokine to regulate HSC homing to the bone marrow [68]. CXCR4 overexpression was found to be a marker for bone metastases in many cancer cells, including breast and prostate cancer, where it facilitates metastatic colonization and survival in the bone microenvironment [69,70]. Huang et al. showed that blocking the SDF-1/CXCR4 axis inhibits the formation of breast cancer skeletal metastasis [71]. Following the homing of DTCs in the bone, they may enter a dormant state that sometimes persists for several decades. Bone metastases can develop in both breast and prostate cancers, in which case they can relapse years after the primary tumor was diagnosed. Based on the finding that CXCL12 expression inhibits cancer cell proliferation, instead maintaining them in a dormant state, Wang et al. suggested that for the development of bone metastasis outgrowth, long-term survival, bone retention and dormancy are needed [63]. Other factors that were found to induce cancer cell dormancy are bone morphogenetic proteins (BMPs), TGFβ2 and growth-arrest-specific protein 6 (GAS6), factors that are also known to regulate HSC quiescence [67]. In a process that is not yet fully understood, dormant cells may be reactivated, after which they can initiate metastatic outgrowth in the bone microenvironment. 

Bone metastases are categorized as either osteolytic or osteoblastic (osteosclerotic). Osteolytic lesions are characterized by excessive bone loss, while osteoblastic metastases typically feature abnormal levels of new bone formation. Both processes are typically present, and one ultimately tips the balance toward either bone loss or gain, or they coexist in a mixture of the two types of lesions [72]. Although osteolytic metastasis is the dominant type of breast cancer, up to 25% of patients are estimated to have osteoblastic lesions [54]. The crosstalk between breast cancer (BC) cells and the bone microenvironment results in a “vicious cycle” of bone destruction and increased tumor growth in the bone [73]. BC cells that survive and metastasize to the bone secrete different molecules, such as parathyroid hormone-related protein (PTHrP), TNFα, MMP1, etc. As a result, osteoblasts start producing IL-6 and RANKL, which, in turn, stimulate osteoclast differentiation and activity. Higher osteoclast activity leads to osteolytic bone destruction and the release of the growth factors TGFβ, IGF1 and PDGF, which ultimately enhances tumor cell survival and growth and drives more bone destruction [74,75,76]. Osteoblastic bone metastasis occurs mainly in prostate cancer. In this type of bone metastasis, osteoblasts adjacent to metastatic tumor cells are stimulated and may be measured by osteocalcin and alkaline phosphatase levels [72,77]. A few factors were found to have a role in osteoblastic metastasis, such as endothelin-1 (ET-1) and prostate surface antigen (PSA). ET-1 was found to stimulate osteoblast proliferation and bone formation, and its expression is upregulated in prostate cancer and is strongly correlated with osteoblastic bone metastasis occurrence [63,64]. Activated PSA in the bone microenvironment was found to enhance tumor growth via the elevation of Wnt signaling and the upregulation of RNKL, TGF-β and RUNX-2, which promote osteoblastic differentiation and activity. However, a better understanding of the process and the identification of the other factors that affect the osteoblast–osteoclast balance in bone metastases are needed [63,78]. 

## 4. Wnt Signaling in Bone Metastasis 

Wnt signaling has emerged as a vital mediator of a variety of physiological and pathological processes, including cancer development and bone metastasis [2,12,14]. Alterations in Wnt signaling thus assume a pivotal role in various cancer types. When Wnt and its downstream effectors are deregulated, cancer initiation, growth, metastasis and drug resistance are promoted. Wnt signaling pathways play crucial roles in maintaining cancer stem cells in various cancer types. Previous studies have demonstrated that cancer stem cells are more resistant to chemotherapy and radiotherapy. This may be due to their higher expression of multidrug resistance genes, including ATP-binding cassette, and antiapoptotic proteins such as MYC (Wnt target gene). Therefore, cancer stem cells can survive and lead to cancer relapse and contribute to tumor metastasis development [79].

**Figure 2:**
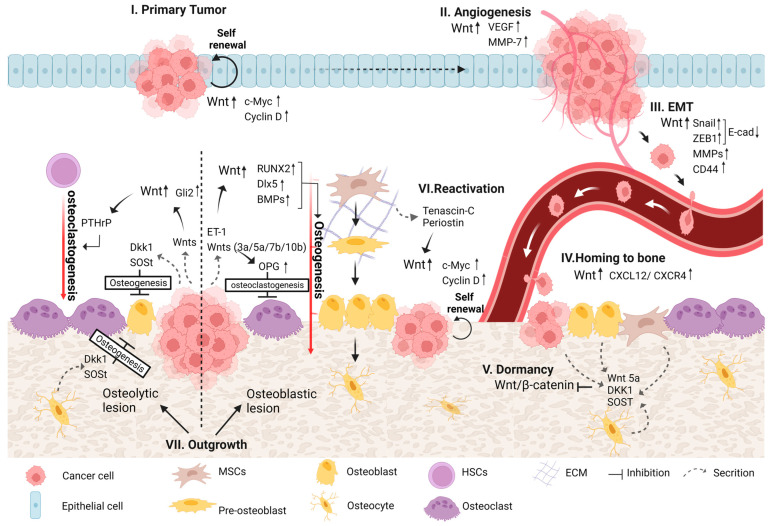
Wnt signaling in the development of bone metastases. The roles of Wnt signaling pathways in multiple steps during the process of bone metastasis formation and progression. The primary tumor progresses (I, II). EMT is taking place, and invading cells depart from the primary tumor (likely breast, prostate, lung, multiple myeloma, etc.) and intravasate the vasculature (III). Under the effects of Wnt and other attractants, the cells home and attach to bone marrow niches (IV). Interactions within the bone marrow compartments may drive long-term dormancy (V). Following a myriad of cues, quiescent cells are reactivated (VI), and osteoblastic or osteoclastic outgrowth may cause bone deformation and lesions. The Wnt-related landscape of these processes is portrayed in detail in the text below.

Wnt pathways were previously reported to be involved in different forms of bone metastases that originate mainly from multiple myeloma, breast, prostate, and lung cancers [80,81,82,83]. In addition, Wnt signaling was shown to regulate different steps during the process of bone metastasis development, from primary tumor progression to the induction of osteolytic/osteoblastic bone metastases [81,84] (Figure 2). 

### 4.1. Primary Tumor Development

The aberrant regulation of Wnt signaling may lead to neoplastic proliferation and subsequent tumor formation and growth [3]. The activation of the canonical Wnt pathway contributes to tumor initiation and progression in several cancer types, including those arising in bone and different carcinomas that form in epithelial tissues, such as osteosarcoma, colon, breast, prostate, multiple myeloma and others [14,18,85,86]. Among the explanations proposed for elevated canonical Wnt signaling are loss-of-function mutations in APC (part of the β-catenin destruction complex), mutations in β-catenin that prevent it from being marked for degradation, and the overexpression of Wnt ligands and receptors that lead to the accumulation of high cytoplasmic levels of β-catenin [10]. Moreover, genes transcribed by the β-catenin/TCF complex, including but not limited to cyclin D1 and c-Myc—which are involved in the cell cycle and thus regulate cell growth and proliferation—were reported to be inappropriately activated [3]. In addition, c-Myc transcription was also found to be upregulated by the non-canonical Wnt/Ca^2+^ pathway through NFAT, which caused increased cell proliferation [72]. 

### 4.2. Angiogenesis 

Angiogenesis is one of the hallmarks of cancer proposed by Hanahan and Weinberg (2000, 2011). Capillary blood vessels are normally present in all tissues to supply them with nutrients and oxygen and to eliminate carbon dioxide and metabolic waste. While normal vasculatures in healthy adults are mainly quiescent, in cancer, the angiogenic process includes a switch from quiescent to sprouting vessels by tipping the balance between pro- and anti-angiogenic molecules via altered gene transcription and the control of factor bioavailability, the main triggers for which are metabolic stress, hypoxia and mechanical stress in the tumor [52,53,87]. The new blood vessels that sprout within the solid tumor are essential to cancer progression, in that they provide an escape route for invading tumor cells and allow cancer cells to recruit circulating bone-marrow-derived cells that amplify tumor angiogenesis [88]. Wnt pathways play an important role in angiogenic blood vessel remodeling in both normal and cancerous conditions. Wnt/β-catenin signaling was shown to regulate the transcription of vascular endothelial growth factor (VEGF), which stimulates the critical stages of endothelial proliferation, migration and survival and plays a crucial role in physiological and pathological angiogenesis [89]. A mutation in APC that renders β-catenin constitutively active may lead to the overexpression of VEGF in colon cancer [90]. In addition, previous studies demonstrated that targeting Wnt signaling led to angiogenesis inhibition in breast cancer [91,92,93]. Furthermore, Wnt pathways were found to regulate the transcription of the angiogenic factors interleukin-8 and MMP7, thereby enhancing angiogenesis in pancreatic, prostate and breast cancers [94,95,96]. 

### 4.3. EMT

During the EMT process, cancer cells acquire a mesenchymal phenotype and even stem-cell-like features that enable them to migrate, invade and eventually metastasize to different organs, and therefore, it is considered one of the principal routes for tumor progression [52,97]. The activation of Wnt signaling may induce EMT in many types of cancer—for example, breast, prostate, ovarian, colorectal, uterine carcinomas and others—wherein it facilitates cell motility and invasiveness [98,99,100]. The Wnt/β-catenin pathway was found to activate the transcription factors ZEB and Snail, which repress the expression of E-cadherin [101]. In addition, β-catenin plays a pivotal role in the regulation of cell adhesion during its binding to and linking of the cytoplasmic domain of type I cadherins to the actin cytoskeleton [102]. During the EMT process, the loss of E-cadherin expression or β-catenin phosphorylation alters its distribution within the cells, which causes the cells to acquire an elongated shape and the ability to invade the ECM [103]. 

There is growing evidence, however, that β-catenin activation may occur through cues other than those involved in Wnt signaling, for example, the epidermal growth factor receptor (EGFR) in melanoma [20] and the androgen receptor (AR) in prostate cancer, which was found to be a target of Wnt [104]. Other Wnt target genes that are known to have a role in the EMT process are matrix metalloproteinases (MMPs) and CD44. The upregulation of different members of the MMP family (e.g., MMP2, MMP3, MMP7 and MMP9) in prostate, gastric and breast cancers, among others, leads to the acquisition of an invasive phenotype [104,105,106]. The cancer stem cell marker CD44 was shown to play a role in EMT induction and maintenance in prostate, breast, lung and head and neck cancers [107,108,109]. Taken together, these studies show that Wnt plays a pivotal role in the EMT processes that are needed to initiate metastasis and create DTCs.

### 4.4. Homing of DTCs to Bone

Working in concert with chemo-attractive and adhesion molecules, Wnt signaling has been implicated in the homing of tumor cells to bone. A previous study revealed that high miR-218, a micro-RNA induced during osteoblast differentiation, positively correlated with the robust stimulation of Wnt signaling in bone-homing breast cancer cells [110]. Wnt signaling was also shown to promote the attachment of circular prostate cancer cells to osteoblasts via the secretion of Wnt-induced secreted protein-1 (WISP-1). Following WISP-1 secretion, the expression of α4β1 integrin, which is crucial for prostate cancer cell/osteoblast attachment, is initiated [111]. The non-canonical Wnt ligand Wnt5a, which may regulate cellular motility via the Wnt-PCP pathway, was shown to promote the homing of prostate cancer cells to bone [84,111]. As mentioned before, the overexpression of the CXCL12/CXCR4 complex was found to be a marker for bone metastases in many cancer types [69,70]. In addition, Wnt signaling has been shown to regulate the CXCL12/CXCR4 axis. β-Catenin binding to the promoter region of CXCL12 via a LEF/TCF-binding site increases its expression [112]. In addition, under hyaluronan-rich conditions, the binding of the Wnt target gene CD44 to this complex leads to its activation [113]. 

### 4.5. Colonization of DTCs in Bone

Following DTC homing in the bone, DTCs either enter a dormant state or divide rapidly to create a secondary tumor in the bone. Both processes depend on the secretion of different factors within the bone microenvironment that stimulate different pathways in tumor cells [66]. These signals are similar to those that act on normal bone-resident stem cells present in the bone niches [114]. 

#### 4.5.1. Dormancy

Accumulating studies have shown that the downregulation of the canonical Wnt pathway contributes to cancer cell dormancy. The mechanisms by which Wnt and other key signaling pathways support cancer dormancy, however, remain to be elucidated [84,115]. The non-canonical Wnt ligand Wnt5a, secreted by osteoblasts in the endosteal niche, induces and maintains prostate cancer cell dormancy via the activation of non-canonical Wnt signaling, resulting in the suppression of the canonical Wnt pathway [115]. Previous studies demonstrated that the inhibition of canonical Wnt signaling may lead to a switch by dormant colorectal cells to senescence [84,116]. Interleukin-23 receptor signaling was found to mediate cancer cell dormancy via the Wnt/Notch pathway in human esophageal squamous carcinoma cells [117]. In addition, the Wnt inhibitors DKK1 and SOST secreted by MSCs, osteocytes or cancer cells were found to induce slow cycling or a dormant state in multiple myeloma, breast, lung and liver cancers [118,119,120,121]. In contrast, as DKK1 is a Wnt target molecule, Kim et al. suggested that Wnt activation may be essential for cancer dormancy by upregulating DKK1 [112]. 

#### 4.5.2. Reactivation and Outgrowth

At some point, cancer cells can escape dormancy and reinitiate proliferation to form colonies in the bone via mechanisms that are not fully understood. This may be achieved by the activation of autocrine transcription within cancer cells and/or by paracrine stimulation from cells in the bone microenvironment that promote cell cycle pathways [112]. To date, the precise interactions and molecules that are involved in the process, however, are poorly understood [122]. The stem cell niche was identified as playing an important role in the reactivation of cancer cells. The presence of the ECM proteins tenascin-C and periostin in this niche supports the outgrowth of cancer micro-metastases by activating both the Wnt and Notch signaling pathways. These pathways activate the cell cycle via cyclin D1 and Myc, resulting in the self-renewal of cancer cells. Other research showed that in BC cells, bone-marrow-derived IL1β induces intracellular NFkB and CREB signaling, leading to autocrine Wnt signaling that results in BC cell colony formation in the bone. Furthermore, inhibition of this pathway hampered BC cell colonization in the bone [123]. In addition, the Wnt/TCF target genes LEF1 and HOXB9 were shown to promote the colonization of lung adenocarcinoma cells in bone and the brain [124]. Taken together, these studies show that Wnt has a prominent role in the reactivation of dormant cells via the upregulation of the cell cycle, which leads to metastatic outgrowth and bone metastatic colonization. 

#### 4.5.3. Osteoblastic and Osteolytic Lesions

During the development of bone metastasis and the outgrowth of the secondary tumor, the balance between bone formation and resorption is disrupted. Bone metastases induce bone lesions that can range from bone-forming (osteoblastic) to bone-destructive (osteolytic) lesions. In general, osteoblastic lesions are associated with high osteoblast activity and variable osteoclast activity, while in osteolytic lesions, the activity of osteoblasts is typically reduced, and that of osteoclasts is amplified. These changes in the microenvironment of the tumor lead to the further progression of the secondary tumor [125]. The type of bone lesion formed is determined by the type of cancer cells and is affected by factors from the bone microenvironment and/or the tumor cells themselves that stimulate different cues. However, further study is needed to understand the relative contribution of autocrine factors released by cancer cells versus that of paracrine factors released from the bone microenvironment vis-à-vis the type of bone lesion formed [33]. One such cue is Wnt signaling, as it plays an important role in regulating both bone homeostasis and the different tumorigenic properties of tumor cells. 

##### Osteoblastic Lesion

The crucial role of Wnt signaling in osteogenesis regulation, described in detail in Section 2, indicates that Wnt signaling is likely a key driver, together with other cues, of osteoblastic lesion formation. Previous studies showed that prostate tumor cells secrete different Wnt ligands, such as Wnt3a, Wnt 5a, Wnt7b and Wnt 10b, stimulating both the canonical and non-canonical pathways and leading to osteoblast differentiation and new bone mineralization [125,126,127]. The canonical Wnt ligand Wnt3a and the non-canonical ligand Wnt5a were shown to induce BMP-4 and -6 expression in prostate cancer and to stimulate osteoblast differentiation that resulted in osteoblastic lesion formation [128,129]. Moreover, the administration of the Wnt inhibitor DKK1 and the BMP inhibitor Noggin to those cells reduced osteoblast differentiation in a synergistic fashion [128]. Both DKK1 and Noggin may be secreted either by cancer cells or by microenvironment cells, such as osteocytes [125,130]. Another study showed that the transfection of both CB-2B and PC-3 prostate cancer cells with DKK1 resulted in a reduction in osteoblastic activity that also led to the formation of osteolytic lesions in CB-2B cells [126]. The non-canonical Wnt ligand Wnt7b produced by prostate cancer cells induced osteoblast differentiation and osteoblastic lesion formation in bone via direct cell–cell interactions between cancer cells and osteoblasts [131]. Activated Wnt signaling leads to the upregulation of miR-218, which, in turn, induces the Wnt pathway by downregulating the Wnt inhibitors SOST and DKK2 and the secreted frizzled-related protein 2 (SFRP2). This positive Wnt signaling loop affects bone marrow stromal cells (BMSCs) to support both the commitment to and progression of osteogenesis in breast cancer [110]. In prostate cancer, T-box2 (TBX2) was found to promote the transcription of Wnt3a, which, together with the Wnt effector TCF4, is highly overexpressed in osteoblastic bone metastasis [132]. ET1 secreted by cancer cells in the bone microenvironment, known as a major mediator of bone deposition, was found to downregulate the expression of DKK1 and to upregulate the expression of Wnt5a [133,134]. The canonical Wnt pathway was reported to have a significant effect on osteogenesis in breast cancer. This effect was further increased by the stimulation of Wnt by ET1 in MCF7 and MDA-MB-231 BCCs cultured on a three-dimensional (3D) nano-clay scaffold [135]. Overall, these findings strongly indicate that Wnt signaling is crucial for osteogenesis and the formation of osteoblastic bone metastasis. Furthermore, Wnt inhibition leads to a reduction in osteogenesis and may result in a switch from osteoblastic to osteolytic lesion formation.

##### Osteolytic Lesion 

An osteolytic lesion is characterized by enhanced osteoclast activity and reduced osteoblast activity, and together, they cause massive bone destruction and initiate the “vicious cycle” of cancer. Tumor cells secrete different factors that stimulate osteoclast activity, and prominent among them is PTHrp. Wnt activation has been reported to increase PTHrP expression via Gli2 regulation, leading to bone distraction both in MDA-MB-231 BCCs and in RWGT2 non-small-cell lung (NSCL) cells in vivo [136]. In breast cancer, miR-218-5p activates Wnt signaling. This activation leads to the expression of bone sialoprotein, osteopontin, CXCR-4 and PTHrP and the induction of osteolytic metastasis [137]. The canonical Wnt pathway regulates the expression of the transcription factor TWIST via HIF-1α activation [138], a factor that was found to promote the development of osteolytic bone metastasis via a mechanism dependent on miR-10b in breast cancer [139]. In addition, tumor cells secrete and/or stimulate the secretion of different factors that inhibit osteoblast differentiation and activity, among which are different Wnt antagonists. The Wnt antagonist and Wnt target gene DKK1 are known to enhance osteolytic bone lesion formation by downregulating osteoblast activity and upregulating osteoclastogenesis. Expressed in multiple myeloma, breast, lung and prostate cancers, this Wnt antagonist was first reported to promote osteolytic lesions in multiple myeloma [125,140]. DKK1 overexpression in MDA-MB-231 BCCs led to an increase in osteoclasts via the downregulation of Wnt signaling within osteoblasts, resulting in significantly increased osteolysis with enhanced tumor proliferation in vivo [141]. Clines et al. showed that DKK1 expression was relatively high in cells that generate osteolytic lesions, such as MDA-MB-231 and PC3, in comparison with cancer cells that produce osteoblastic or mixed lesions. Despite DKK1 expression, Wnt signaling was found to be active in these cells due to the downregulation of DKK1 receptors, a phenomenon that was suggested as a mechanism of DKK1 resistance in osteolytic-lesion-forming tumor cells [142]. In addition, the overexpression of DKK1 in small-cell lung cancer SBC-3 cells was shown to promote osteolytic bone metastasis in vivo [143]. The Wnt antagonist SOST was also reported to enhance cancer-induced osteolytic bone metastasis, and its inhibition prevented osteolytic lesion formation in MDA-MB-231 and MCF7 xenograft models [144]. Another two Wnt antagonists, Wnt inhibitory factor1 (WIF1) and SFRP4, are overexpressed in a mouse model of “breast cancer-specific osteolysis”. These antagonists led to the downregulation of Wnt2 and 8b ligands, resulting in suppressed bone formation and increased bone resorption [145]. Overall, these findings strongly suggest that Wnt pathways have a role in osteoclastogenesis. In addition, the secretion of Wnt antagonists by tumor cells targets mainly bone cells, while tumor cells might resist them. These antagonists are essential to the reduction in osteogenesis and the induction of bone destruction.

#### 4.5.4. Transmission of Bone Pain

In addition to the involvement of Wnt pathways in every significant phase during bone metastasis development, Wnt signaling also takes part in the transmission of bone pain [146]. Wnt5b and the co-receptor RYK were upregulated in the sensory dorsal root ganglia of bone-tumor-bearing mice and therefore were assumed to participate in the transduction of pain due to bone cancer [147]. In another study, Wnt3a was significantly increased in the spinal cord dorsal horn and colocalized with neurons and astrocytes in a mouse model of bone cancer pain [148,149]. 

## 5. Therapeutic Opportunities and Future Perspectives

Wnt signaling comprises important pathways that regulate many pivotal cellular activities, such as fate decisions, proliferation, migration, and differentiation. The pathways are activated mainly during embryonic development and within the context of stem cell maintenance and tissue homeostasis. As we describe in this review, both the canonical and non-canonical pathways control postnatal bone homeostasis, which is maintained by the delicate balance between bone formation and resorption by osteoblasts and osteoclasts. In recent years, Wnt signaling has been shown to have a role in bone biology, and aberrant Wnt signaling activity/regulation has been understood to be a possible cause of different bone pathologies and to occur in the development of various malignancies. Wnt signaling was also found to be involved in the multi-step process of bone metastasis, providing tumor cells with the ability to migrate, invade and home to the bone, where they survive and thrive within its microenvironment. Furthermore, despite growing evidence, the role that Wnt signaling pathways play in this complex process remains to be elucidated. We believe that a better understanding of these pathways will provide insights into tumor biology and behavior that may later prove to be fertile ground upon which to base potential therapeutics for bone metastasis. Targeting Wnt signaling and its components, therefore, may provide a potential therapeutic option for various cancer types in combination with other therapies [150]. A particularly promising route to target Wnt signaling is via the inhibition of their production or secretion by, for example, selectively inhibiting the activity of porcupine (PRCN), a transmembrane protein located in the endoplasmic reticulum. It adds a palmitoyl group to Wnt ligands that is essential for their signaling ability and that is required for the secretion of all Wnt ligands [2,151]. Previous studies showed that PRCN overexpression correlates with Wnt signaling activity in gastric and lung cancers. A few molecules were found to inhibit PRCN, such as inhibitors of Wnt production (IWPs), LGK974 and ETC-195 [24,152]. In their review, Shah et al. suggest that Porcupine inhibitors might be emerging therapeutics that slow down bone metastasis by possible underlying mechanisms such as suppressing EMT, regulating osteoblast differentiation, etc. [153]. Another strategy for Wnt inhibition is to inhibit Wnt receptors, such as Fz or LRP, by, for example, using antibodies against the Fz receptor, OMP-18R5, OMP-54F28 and OTSA 101, that compete with Fz for Wnt ligands [24,150]. The agents 3289–8625, FJ9 and NSC668036 block the Fz-DVL interaction, resulting in the inhibition of signal transduction [142]. The inhibition of Tankyrases 1 and 2, which target Axin for proteasomal degradation, leads to increased Axin levels that activate the β-catenin destruction complex. This could be achieved by using the agents Xav939, IWR1, NVP-TNKS656 and JW74 [14,15,154]. ICG-001, PFK115-584 and CGP049090 are examples of compounds that inhibit the transcription complex β-catenin/TCF and may serve as a promising strategy for targeting Wnt downstream effectors [154,155]. Previous studies revealed that ICG-100 reduces the cancer stem-like properties of carcinoma cells [156]. In addition, this small molecule was found to inhibit osteogenesis in rats [157]. These findings imply that ICG-100 may serve as a potential therapeutic option for patients with osteoblastic bone metastasis. As described in this review, DKK1 expression is relatively high in cells that generate osteolytic lesions. Several studies showed that neutralizing antibodies for DKK1, SFRP1 and SOST increase bone mass and reduce osteolysis in animal models [158]. In addition, a DKK1-neutralizing antibody was shown to suppress multiple myeloma growth and tumor-induced bone resorption in mice [159]. To date, no Wnt inhibitors have made it into the clinic, probably because Wnt signaling plays a pivotal role in the normal processes of other cells and in tissue homeostasis and not only in the progression of cancer cells, particularly in bone, where Wnt pathways play a key regulatory role in bone homeostasis, as described in this review. A promising strategy, therefore, may be to target inhibitors to the afflicted tissue/cancer cells directly [24]. Several nanoparticle systems for the delivery of Wnt inhibitors have been developed in the past few years. For example, a previous study showed that breast cancer cells that were treated with peptide-based nanoparticles (NPs) loaded with the PRCN inhibitor IWP-2 showed a significant decrease in tumorigenic capacity, which was attributed to improved IWP solubility, cellular uptake and efficacy [160]. In another research zinc-oxide NPs combined with the β-catenin inhibitor ICG-001 led to a synergistic inhibitory effect on osteosarcoma progression; however, the effect of these NPs on bone cells was not examined in this study [161].

Sun et al. describe the role of Wnt signaling in the bone microenvironment as a “double-edged sword”, as it stimulates cancer progression and, on the contrary, contributes to generating anti-tumor capabilities via its co-receptor LRP5 [162]. Recent studies revealed that osteocytes, osteoblasts and MSCs that overexpress LRP5, and β-catenin hampered tumor progression and bone loss. Therefore, this was suggested as a possible novel option for targeting, hence protecting bone from cancer progression [162,163,164]. 

In addition, due to the complexity of the physical, chemical, and topographical landscapes of bone tissue, adequate 3D in vitro models for the study of bone metastases would be highly beneficial to the field, where they would help researchers develop a better understanding of the disease and efficiently scan for new therapeutic targets. Successful 3D culturing models should closely mimic several aspects of in vivo bone tissue behavior, such as tissue architecture and biophysical and biochemical factors. They must also be amenable to the culturing of heterogeneous cell populations and support interactions between different cell types. Lastly, they should also facilitate the formation of new 3D tissue by enabling sufficient nutrient supply to and effective waste removal from the cells in the tissue. To date, there have been several studies focusing on the development of suitable 3D in vitro models for studying bone metastases. These models include scaffolds of natural or artificial biomaterials, microfluidics, bioreactors, microfabrication, and implantable niches [165,166,167,168,169]. For instance, a dense 3D collagen hydrogel was shown to provide a suitable model for studying the interactions between breast cancer cells and osteoblasts within a 3D collagenous microenvironment [170]. The naturally derived solid scaffold, chitosan, incorporated with hyaluronic acid, was found to be suitable for natural tissue imitation [171]. Other 3D scaffolds produced from the exoskeletons of the marine invertebrates *Porites lutea* and *Millepora dichotoma* were shown to provide a suitable microenvironment for culturing various cells (e.g., fibrosarcoma, MSCs, chondrocytes, adipocytes and neuron cells) [172,173,174,175,176,177]. We submit that 3D in vitro models will enable the study of pivotal processes during the development of bone metastases while controlling different factors and the level of complexity.

## Data Availability

Not applicable.
